# “Let’s Dry up and Survive Together”: Is Anhydrobiosis in Two *Paramacrobiotus* Species (Tardigrada) Associated with a Specific Microbiome Community?

**DOI:** 10.3390/ijms27125256

**Published:** 2026-06-10

**Authors:** Monika Mioduchowska, Pushpalata Kayastha, Magdalena M. Bartylak, Edyta Konecka, Bayu Brahmantio, Julita Mackiewicz, Wojciech Przybyszewski, Aleksandra M. Naczk, Marcin Górniak, Jason Pienaar, Edyta Fiałkowska, Łukasz Kaczmarek

**Affiliations:** 1 Department of Evolutionary Genetics and Biosystematics, Faculty of Biology, University of Gdańsk, Wita Stwosza 59, 80-308 Gdańsk, Poland; juli.mackiewicz@gmail.com (J.M.); w.przybyszewski.717@studms.ug.edu.pl (W.P.); marcin.gorniak@ug.edu.pl (M.G.); 2 Department of Biology, University of North Carolina at Chapel Hill, Coker Hall, 120 South Road, Chapel Hill, NC 27599, USA; pushpalata.kayastha@gmail.com; 3 The Institute of Environment & Department of Biology, Florida International University, Miami, FL 33199, USA; jpienaar@fiu.edu; 4 Department of Animal Taxonomy and Ecology, Adam Mickiewicz University in Poznań, Uniwersytetu Poznańskiego 6, 61-614 Poznań, Poland; maggor12@st.amu.edu.pl (M.M.B.); kaczmar@amu.edu.pl (Ł.K.); 5 Department of Microbiology, Adam Mickiewicz University in Poznan, Uniwersytetu Poznańskiego 6, 61-614 Poznań, Poland; edyta.konecka@amu.edu.pl; 6 Department of Computer and Information Science, Linköping University, SE-581 83 Linköping, Sweden; bayu.brahmantio@liu.se; 7 Department of Plant Taxonomy and Nature Conservation, Faculty of Biology, University of Gdańsk, Wita Stwosza 59, 80-308 Gdańsk, Poland; aleksandra.naczk@ug.edu.pl; 8 Institute of Environmental Sciences, Faculty of Biology, Jagiellonian University, Gronostajowa 7, 30-387 Cracow, Poland; edyta.fialkowska@uj.edu.pl

**Keywords:** water bears, desiccation, bacterial 16S rRNA gene, NGS, host-microbiota interactions, COGs

## Abstract

This study reports, for the first time, changes in the microbiome community associated with anhydrobiosis in two tardigrade species of the genus *Paramacrobiotus*. To identify bacteria linked to the anhydrobiosis phenomenon and to track microbiome changes under anhydrobiotic stress, next-generation sequencing of bacterial 16S rRNA genes was conducted. Microbiome profiling was performed across various developmental and physiological stages of tardigrades, including: eggs; active adult specimens (both before and after 7, and 120 days of anhydrobiosis, referred to as short- and long-term anhydrobiosis, respectively); specimens in the desiccated tun stage; dead specimens following long-term anhydrobiosis (no dead specimens were observed after short-term anhydrobiosis); and the culture medium. It was shown that the microbiome community varied among stages, with high stage-specificity. Several bacterial genera were identified that may assist the host during anhydrobiosis, potentially through biofilm formation and by supporting stress-protective mechanisms such as heat shock protein expression and trehalose synthesis in eggs and tuns. These findings reveal that microbiota may contribute to anhydrobiotic survival in tardigrades, providing novel insights into host–microbe interactions under extreme environmental stress.

## 1. Introduction

Anhydrobiosis is the ability of an organism to lose more than 90% of its intracellular water, mount a regulated response to produce various cellular protectants, enter a state of suspended metabolism, and then to regain normal biological activity upon rehydration. It occurs, e.g., in many bacteria, lichens, bryophytes, tardigrades, rotifers, nematodes, yeasts and desiccation-tolerant plants (e.g., refs. [1,2] and citations therein). Despite the occurrence of anhydrobiosis in both eukaryotic and prokaryotic organisms, some differences have been observed. First, prokaryotic anhydrobionts appear to maintain detectable levels of metabolism in the desiccated stage, although higher-level functions such as reproduction and motility are rapidly inhibited. Most changes occur at the gene expression level, but many prokaryotes also seem to switch to a different form of slow metabolism when desiccated rather than shutting down entirely. Unsurprisingly, amongst single-celled eukaryotes, yeasts are by far the best studied when it comes to anhydrobiosis [3], as they have a long history of being used in their dehydrated stage as starter cultures for bread, beer, wine, etc., and because they are the model organism for the eukaryotic cell. Yeast studies have shown that practically all cellular and organelle components undergo structural and functional changes to deal with the mechanical, structural and oxidative damage associated with desiccation [4]. Among multicellular eukaryotes, water bears (Tardigrada) are a perfect model to investigate mechanisms responsible for successful anhydrobiosis (e.g., ref. [5]). Limnoterrestrial tardigrades inhabit mosses, lichens, soil or leaf litter, where they reside in transient, interstitial films of water, a characteristic shared with intertidal marine tardigrades. One of the best-known attributes of such tardigrades is their ability to enter a stage of suspended animation and metabolism called cryptobiosis (of which anhydrobiosis is a subset), which enables them to withstand numerous harsh environmental conditions, including complete desiccation, very low and high temperatures, space vacuum, extremely high and low pressure, and even high doses of ionizing and UV radiation [6]. Tardigrade anhydrobiosis results in up to 95% reduced cellular water in some species and can be maintained for more than a decade [7]; however, the specific mechanisms responsible for these abilities are still poorly understood.

All multicellular organisms harbour their own specific microbiome that exerts various effects on their biology [8]. While tardigrade survival strategies are receiving plenty of recent attention, their microbiomes remain poorly understood [9,10,11,12,13,14,15,16,17,18]. Nevertheless, recent advancements in tardigrade microbiome research, based on 16S rRNA gene amplicon sequencing, have enabled detailed analyses revealing unique microbiome compositions shaped by both environmental factors and host species. Vecchi et al. [9] analyzed six limnoterrestrial tardigrade species and found Proteobacteria and Bacteroidetes dominant, with profiles distinct from surrounding habitats and varying across species. They also noted that laboratory culturing alters microbiomes and detected potential Rickettsiales endosymbionts, which can influence reproduction. Kaczmarek et al. [11] studied *Paramacrobiotus experimentalis* and confirmed that the tardigrade microbiome was distinct from artificial laboratory environments, with Proteobacteria and Firmicutes being the predominant bacterial phyla. Further contributing to this field, Mioduchowska et al. [13] examined four tardigrade species and found Proteobacteria, Firmicutes and Actinobacteria, along with Rickettsiales and *Wolbachia* in eggs and adults (suggesting vertical transmission). Later, Mioduchowska et al. [14] developed tools confirming *Wolbachia* in *Pam. experimentalis* and *Macrobiotus basiatus*. In a different ecological context, Zawierucha et al. [17] analyzed the microbiota of the *Cryobiotus klebelsbergi*, collected from cryoconite holes. Their study revealed that microbial diversity was higher in cryoconite compared to both fed and starved tardigrades, with specific bacteria like *Polaromonas* sp. being notably prevalent [17]. Additionally, Tibbs-Cortes et al. [16] studied tardigrade microbiomes in agricultural areas and detected distinct tardigrade microbiomes in comparison to their environments, as well as potential phytopathogens and obligate intracellular microorganisms such as *Rickettsia* and *Wolbachia*. In turn, McQueen et al. [15] concluded that host identity influences gut microbiome composition more than environment, with sampled gut communities being less diverse and distinct from environmental microbes.

The microbiome can affect many aspects of invertebrate hosts’ biology, including evolution, development, metabolism, health, behavior and defence against pathogens. Moreover, microbiota-modulated metabolites control immune activity and development, which on the one hand, suggests that they may play a key role in harsh environments survivability and contribute, e.g., to cryptobiotic abilities. It has also been shown that the host’s diet, phylogeny and genetics have important impacts on bacterial communities specifically and microbial composition [19]. It thus appears that microorganisms that are part of the tardigrade microbiome must similarly to their hosts, tolerate extreme environmental stress. However, to date, there is no data that could help explain if the ability for anhydrobiosis in metazoans is enhanced, diminished or unaffected by specific bacterial communities.

Thus, the main aim of this study was to elucidate whether the microbiome or specific bacteria which are part of this microbiome could affect the ability of tardigrades to undergo anhydrobiosis. Two *Paramacrobiotus* species maintained in long-term laboratory cultures were selected for this study due to their experimental tractability and their well-documented ability to undergo anhydrobiosis, which makes them particularly suitable model organisms for studying stress-related microbial associations. In addition, prolonged maintenance under laboratory conditions likely led to the reduction or loss of transient environmental bacteria, resulting in more stable and host-associated microbial communities, which is advantageous for comparative microbiome analyses. The microbial communities of two *Paramacrobiotus* species at different developmental (eggs and adults) and physiological stages (active specimens before and after anhydrobiosis, as well as specimens in anhydrobiosis, so-called tuns) were examined. Next-generation sequencing (NGS) of the bacterial 16S rRNA gene was used to provide insights into the following questions: (i) What bacteria are present in the microbiome community at different developmental and physiological stages, and what is their relative abundance? (ii) What bacteria can survive desiccation and potentially modulate the anhydrobiotic ability of tardigrades? (iii) Does the metabolic profile of the microbiome change during anhydrobiosis which could indicate an impact on the host’s anhydrobiotic capacity?

## 2. Results

### 2.1. General Description of NGS Reads

In total, 42 DNA samples of the tardigrade microbiome and three negative control samples of the medium with rotifers were analyzed (Table S1). Overall, sequencing produced 8,343,570 reads, 98% of which passed quality control, with a range of 20,605–423,293 reads per replicate (Table S2). The sequencing depth was sufficient for microbiome community characterization, as supported by the observed rarefaction curves with Chao1 diversity indices, which reached a plateau (Figure S1, Table S3).

In turn additional negative control samples—DNA extraction reagents and PCR blanks—were included to assess background contamination. Each category was analyzed in triplicate; however, no NGS reads were obtained for one PCR blank replicate as expected for samples with no visible bands on the agarose gel (Figure S2; see also [20,21]). The average number of sequences obtained in the present study was approximately 450,000. Therefore, sequences detected in negative control samples—ranging from 0 to 226 reads in PCR blanks and from 654 to 1537 reads in DNA extraction blanks—represented at most 0.34% of the mean read count. This level corresponds to background noise with no biological significance.

Metagenetic analysis of the 16S rRNA gene showed that 98.718% of reads belonged to bacteria, while the remaining sequences were classified as follows: (i) 0.003% of reads represented Archaea (193 sequences), distributed across the following stages: *Pe*_active—79 sequences, *Pf*_tuns120—59 sequences and *Pf*_dead120—55 sequences; (ii) 1.279% of reads were categorized as “others” (i.e., non-target and unclassified sequences) with the following distribution: fewer than ten sequences were found in the *Pe*_active, *Pe*_active7 and *Pf*_active120 stages; between 11 and 128 sequences were detected in the *Pe*_tuns7, *Pe*_active120, *Pf*_tuns7, *Pf*_tuns120, *Pf*_active and *Pe*_dead120 stages; 455 sequences were found in the *Pf*_dead120 stage; 631 sequences were found in the *Pf*_active7 stage; and 90,263 sequences were found in the *Pe*_tuns120 stage. No “other” sequences were identified in eggs from either *Paramacrobiotus* species. In the case of the medium, 100% of the obtained sequences were classified as bacteria (Table S4).

### 2.2. Microbial Community Composition

**Figure 1:**
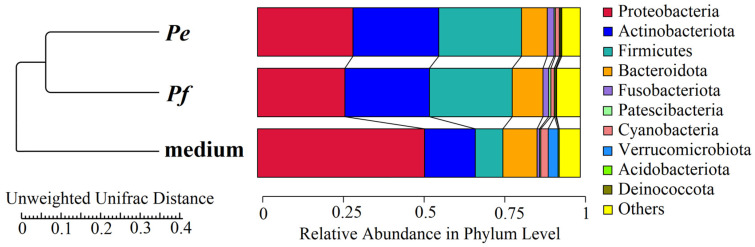
The Unweighted UniFrac Distance Dendrogram, along with the relative abundance of microbial phyla identified in the microbiome communities of *Pam. experimentalis* (described as *Pe*), *Pam. fairbanksi* (described as *Pf*), and their laboratory habitat, i.e., medium with rotifers (described simply as “medium”).

The 45 microbiome communities contained a total of 24 phyla found in tardigrades and 11 phyla identified in the medium. The Unweighted UniFrac Distance Dendrogram revealed the phylogenetic relationship between microbial communities based on the Unweighted UniFrac distance metric and indicated that *Pam. experimentalis* and *Pam. fairbanksi* share similar microbial composition, while the medium exhibits a distinct community structure. The most abundant phyla in the tardigrade microbiome were Firmicutes, Actinobacteriota and Proteobacteria. In contrast, the most dominant phyla in the medium were Proteobacteria, Actinobacteriota and Bacteroidota (Figure 1, Table S5).

All analyses at the genus level for grouped, thrice-replicated samples at each developmental and physiological stage of tardigrades and for the medium as a negative control were performed.

**Figure 2:**
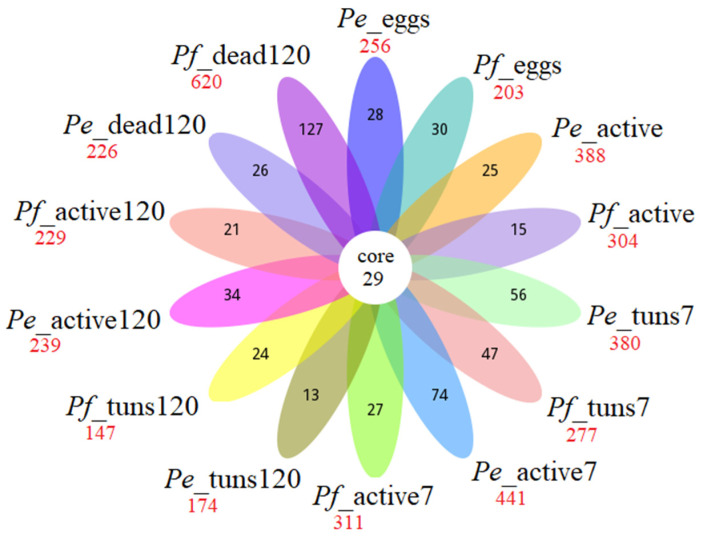
The OTUs petal diagram of each tested stage of the two *Paramacrobiotus* species, including the core microbiome community, unique OTUs (black numbers), and total OTUs (red numbers) at each stage. Abbreviations: *Pe*—*Pam. experimentalis*, *Pf*—*Pam. fairbanski*, 7—short anhydrobiosis (7 days), 120—long anhydrobiosis (120 days).

Figure 2 shows the distribution of OTUs across developmental and physiological stages of the two *Paramacrobiotus* species. The petal diagram displays the core microbiome consisting of 29 OTUs, which were shared among all analyzed stages (including dead individuals after long anhydrobiosis), as well as unique and total OTUs found at each stage. The highest number of total and unique OTUs was observed in *Pf*_dead120 (620 and 127, respectively). In turn the lowest number of total and unique OTUs was found in *Pf*_tuns120 and *Pe*_tuns120 (147 and 13, respectively). Overall, the total and unique OTUs varied across tardigrade stages. In the medium, between 95 and 118 OTUs were detected (Tables S2 and S5–S11).

The following taxa of the bacteria were present (at different taxonomic levels) in the core microbiome:

- Family level: Chitinophagaceae, Neisseriaceae;
- Genus level: *Acidocella*, *Finegoldia*, *Staphylococcus*, *Streptococcus*, *Peptoniphilus*, *Corynebacterium*, *Lawsonella*, *Anaerococcus*, *Sphingomonas*, *Gemella*, *Varibaculum*;
- Species level: *Corynebacterium tuberculostearicum*, *Cutibacterium acnes*, *Ralstonia pickettii*, *Kocuria rhizophila*, *Paracoccus yeei*, *Micrococcus luteus*, *Moraxella osloensis*, *Actinomyces naeslundii*, *Staphylococcus pettenkoferi*.

**Figure 3:**
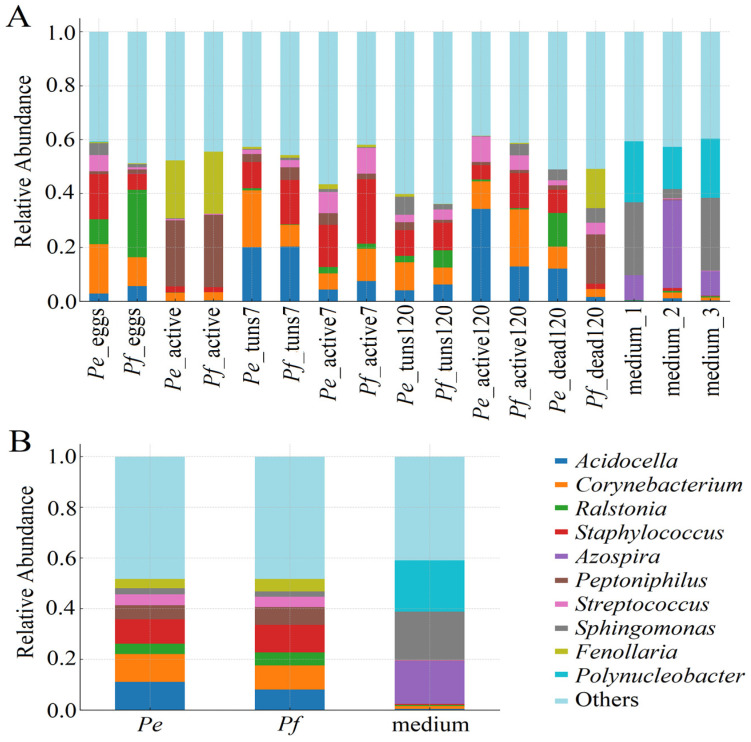
Relative abundance of the ten most abundant bacteria identified to the genus level found in the bacterial communities of *Pam. experimentalis*, *Pam. fairbanksi* and the medium as a negative control: (**A**) microbiome community of tardigrades at different stages associated with anhydrobiosis ability and medium; (**B**) microbiome community of all analyzed samples of *Pam. experimentalis*, *Pam. fairbanksi*, and their respective medium. Abbreviations: *Pe*—*Pam. experimentalis*, *Pf—Pam. fairbanski*, 7—short anhydrobiosis, 120—long anhydrobiosis, medium—*L. inermis* specimens with their medium.

Figure 3A shows the ten most abundant microbial taxa at the genus level, indicating changes across developmental and physiological stages in both *Paramacrobiotus* species and their medium (with three replicates included to demonstrate reproducibility). Additionally, Figure 3B summarizes the relative abundance of the microbial taxa presented in Figure 3A, showing the entire microbiome community identified in *Pam. experimentalis*, *Pam. fairbanksi*, and the medium. Overall bacterial community structure is similar for both *Paramacrobiotus* species and varies according to developmental and physiological stages. All tardigrade microbiomes shared a core set of dominant taxa. In turn less abundant taxa were grouped under an “others” category. The relative proportions of specific genera changed across different developmental and physiological stages. The most frequently observed genera in both *Paramacrobiotus* species were *Acidocella*, *Corynebacterium*, *Ralstonia* and *Staphylococcus*, although their abundance varied across stages.

Overall, the dominant genera at each stage of both *Paramacrobiotus* species were as follows (see also Table S9):

- *Pe*_eggs and *Pf*_eggs: *Ralstonia*, *Corynebacterium* and *Staphylococcus*;
- *Pe*_active and *Pf*_active: *Peptoniphilus*, *Fenollaria*, *Corynebacterium*, *Porphyromonas*, *Negativicoccus*, *Prevotella*, *Finegoldia* and *Ezakiella*;
- *Pe*_tuns7 and *Pf*_tuns7: *Acidocella*, *Corynebacterium*, *Staphylococcus* and *Schlegelella*;
- *Pe*_active7 and *Pf*_active7: *Corynebacterium*, *Staphylococcus*, *Streptococcus*, *Rothia*, *Schlegelella* and *Anaerococcus*;
- *Pe*_tuns120 and *Pf*_active120: *Acidocella*, *Corynebacterium*, *Staphylococcus* and *Acinetobacter*;
- *Pe*_active120 and *Pf*_active120: *Acidocella*, *Corynebacterium* and *Staphylococcus*;
- *Pe*_dead120 and *Pf*_dead120: *Rothia*, *Staphylococcus* and *Peptoniphilus*.

In contrast, the most common genera in the microbiome community of the medium were *Sphingomonas*, *Polynucleobacter*, *Azospira*, *Pseudomonas* and *Limnohabitans*.

### 2.3. Alpha Diversity—Composition Differences Among Bacterial Communities

**Figure 4:**
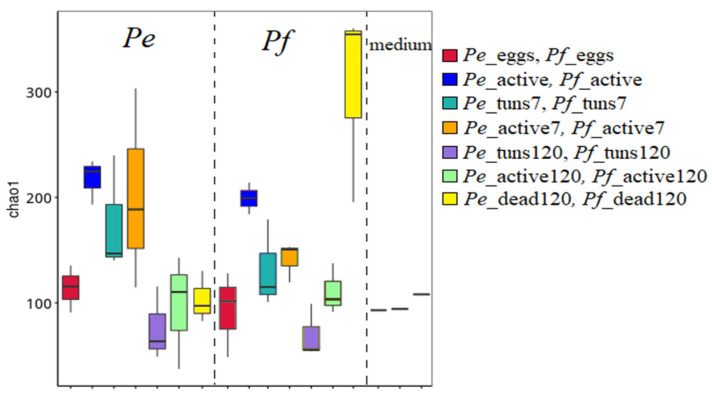
Microbial composition differences among tardigrade developmental and physiological stages as well as their medium measured by the Chao1 index.

Alpha diversity of microbial communities measured by the Chao1 index is presented in Figure 4. The following differences among microbial communities were observed:

- *Pe*_eggs and *Pf*_eggs—relatively low microbial diversity;
- *Pe*_active and *Pf*_active—higher microbial diversity compared to the egg stage;
- *Pe*_tuns7, *Pf*_tuns7, *Pe*_tuns120 and *Pf*_tuns120—both show a clear reduction in microbial diversity compared to active specimens. Furthermore, the 120-day tun stage had a lower median than the 7-day tun stage;
- *Pe*_active7, *Pf*_active7, *Pe*_active120 and *Pf*_active120—partial recovery in microbial diversity. However, diversity remained lower than that of initially active specimens;
- *Pe*_dead120 and *Pf*_dead120—significantly different from all other microbiomes connected with analyzed tardigrade stages and exhibit the highest microbial diversity;
- Medium—significantly different from the microbiome connected with tardigrades.

Differences in the microbiome diversity and composition between the different developmental and physiological stages of tardigrades were tested using PERMANOVA on the distance matrix. Due to the lack of a division into stages in the control sample (medium), this group was excluded from the two-way analysis and was compared separately with the other samples in a one-way ANOSIM analysis. The PERMANOVA revealed a significant difference between *Pam. experimentalis* and *Pam. fairbanksi* in terms of microbiome composition (F_2,43_ = 2.95, *p* < 0.001), as well as between particular developmental and physiological stages (F_7,43_ = 3.32, *p* < 0.001). However, the interaction between examined factors (stages **×** species) was not significant (PERMANOVA, F_14,43_ = 0.02, *p* = 0.999). Conversely, the analysis of similarities (ANOSIM, R = 0.552, *p* < 0.001) demonstrated no statistical differences only among the following pairs of groups (stages): *Pe*_eggs and *Pe*_tuns120, as well as *Pf*_eggs and *Pf*_tuns120 (*p* = 0.49 and 0.42, respectively); *Pe*_active and *Pe*_dead120, as well as *Pf*_active and *Pf*_dead120 (*p* = 0.08 and 0.07); *Pe*_tuns7 and *Pe*_active120, as well as *Pf*_tun7s and *Pf*_active120 (*p* = 0.25 and 0.28); *Pf*_active120 and *Pf*_dead120 (*p* = 0.07).

### 2.4. Potential Bacterial Genera Connected with Anhydrobiosis

#### 2.4.1. Common Bacterial Genera

**Figure 5:**
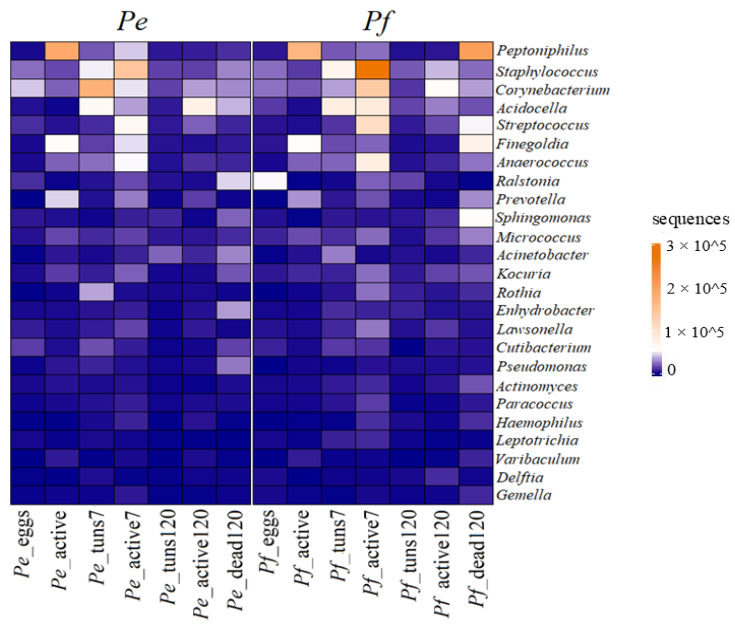
The heatmap with common bacteria identified to genus level in the microbiome community of both *Paramacrobiotus* species at all developmental and physiological stages.

The heatmap indicated the relative abundance of common bacterial genera identified at different stages of two *Paramacrobiotus* species (Figure 5). Overall, the three most dominant common genera were as follows (see also Table S9):

- *Pe*_eggs and *Pf*_eggs: *Ralstonia*, *Corynebacterium* and *Staphylococcus;*
- *Pe*_active and *Pf*_active: *Peptoniphilus, Finegoldia* and *Prevotella*;
- *Pe*_tuns7 and *Pf*_tuns7: *Staphylococcus*, *Corynebacterium* and *Acidocella*;
- *Pe*_active7 and *Pf*_active7: *Staphylococcus*, *Corynebacterium* and *Streptococcus*;
- *Pe*_tuns120 and *Pf*_active120: at this stage all common genera were in low abundance;
- *Pe*_active120 and *Pf*_active120: *Acidocella*, *Corynebacterium* and *Staphylococcus*;
- *Pe*_dead120 and *Pf*_dead120: the abundance of common genera was different for dead specimens of *Pam. fairbanksi* and *Pam. experimentalis*, but it was indicated that death leads to a microbial bloom, with opportunistic bacteria like *Peptoniphilus* and *Staphylococcus* proliferating.

#### 2.4.2. Specific Bacteria for Physiological and Anhydrobiotic Stages

The SIMPER (similarity percentages) analysis identified microorganisms differentiating the developmental and physiological stages of the *Pam. experimentalis* and *Pam. fairbanksi* (Table S12). The presence of *Peptoniphilus*, *Staphylococcus*, *Corynebacterium*, *Acidocella*, *Fenollaria*, *Streptococcus*, *Sphingomonas*, *Finegoldia* and the collective group of microbes described as “others” contributed to the total divergence of more than 50%. However, the overall average dissimilarity was 74.41% for all recognized representatives of the tardigrade microbiome. For *Pam. fairbanksi*, *Peptoniphilus* and *Fennolaria* were particularly distinctive for the active stage; for active7, these were *Staphylococcus*, *Corynebacterium* and *Streptococcus*; for dead120—*Peptoniphilus* and *Fenollaria*. Furthermore, although less pronounced, differentiating characteristics for *Pam. fairbanksi* eggs were observed for *Ralstonia*, *Corynebacterium*, *Staphylococcus* and “others”, and *Acidocella* and *Staphylococcus* for tuns7. In the context of the second species, *Pam. experimentalis*, *Peptoniphilus* and *Fenollaria* were found to be characteristic of the active stage; *Corynebacterium* distinguished tuns7; *Staphylococcus* was evident in active7; tuns120 was defined by “others” and *Acinetobacter*; *Acidocella* was notable in active120; and dead120 was characterized by the presence of “others” and *Ralstonia* and, to a lesser extent, *Acidocella* and *Enhydrobacter*. In the medium, the most differentiating were *Polynucleobacter*, *Azospira* and *Sphingomonas* (Table S9).

The analysis of indicator variables (IndVal%) showed specific phyla that were characteristic to each developmental and physiological stage (Figure S3, Table S13). A preliminary observation revealed a certain dynamic of changes in the composition of the microbiome between the examined developmental and physiological stages for both *Paramacrobiotus* species. *Ferruginibacter* and *Eubacterium* groups were identified as characteristic for the eggs and active stages of *Pam. fairbanksi*, respectively. With a slightly lower frequency, *Sanguibacter-Flavimobilis* and *Aurantisolimonas* were recognized as characteristic for tuns7; for active7—*Schlegelella* and *Kineococcus* were identified; *Vibrionimonas* for tuns120; *Massilia* for active120; and for dead120—*Ornithinimicrobium*, *Cereibacter* and *Acidiphilium* were recorded. In turn, characteristic phyla were also recorded for the individual stages within *Pam. experimentalis*: Rikenellaceae group and *Ottowia* for active; for tuns7—*Rahnella*, *Serratia*, *Aquabacterium*, *Lactobacillus*; for active7—*Moraxella* and *Gardnerella*; and *Microbacterium* for dead120. Statistically significant features are highlighted in the boxes (*p* < 0.05), and the warmer the color, the more indicative the feature. In the medium, the most characteristic representatives of the microbiome in terms of indicators were: *Verrucomicrobium*, *Legionella*, *Peredibacter*, *Lacunisphaera*, *Edaphobaculum*, *Limnohabitans*, *Polynucleobacter*, *Hydrogenophaga*, *Chitinimonas*, *Azosporia* and *Sphingopyxis*.

Overall, there were identified genera in all developmental and physiological stages of *Paramacrobiotus* species that were not detected in the medium, e.g., *Haemophilus*, *Delftia*, *Gemella*, *Leptotrichia*, *Acidovorax* and *Acinetobacter*. Moreover, only in dead specimens, *Aureimonas* and unidentified_67-14 were detected (Table S9).

**Figure 6:**
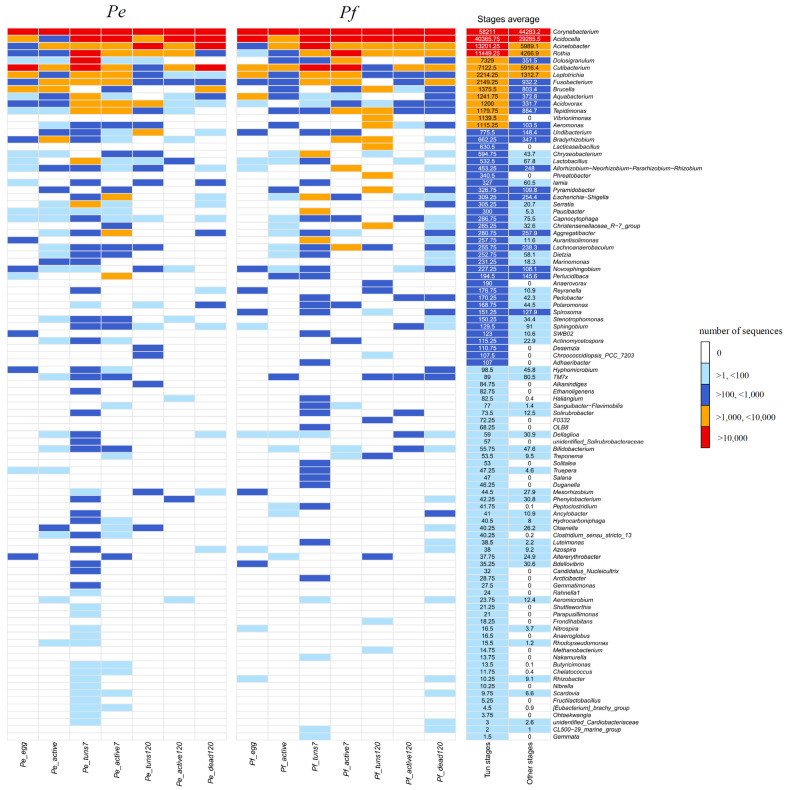
Bacterial genera identified in the microbiome communities of two *Paramacrobiotus* species that showed a higher number of sequences in the tun stages compared to other developmental and physiological stages.

Interestingly, tun stage was characterized by different microbiome composition than observed in other stages. Patterns of bacterial genera distribution clearly showed that there were genera uniquely found only in tuns, i.e., *Vibronimonas*, *Lacticaseibacillus* and *Phreatobacter*. In turn, genera such as *Paucibacter*, *Nocardia*, *Streptococcus* and *Chryseobacterium* showed a notable presence in the tun stage but were rare in other stages. Moreover, many genera were more abundant in tuns than in other stages, e.g., *Serratia*, *Corynebacterium*, *Acinetobacter*, *Cutibacterium*, *Pseudomonas*, *Micrococcus* and *Brevibacterium* (Figure 6).

### 2.5. Functional Prediction

**Figure 7:**
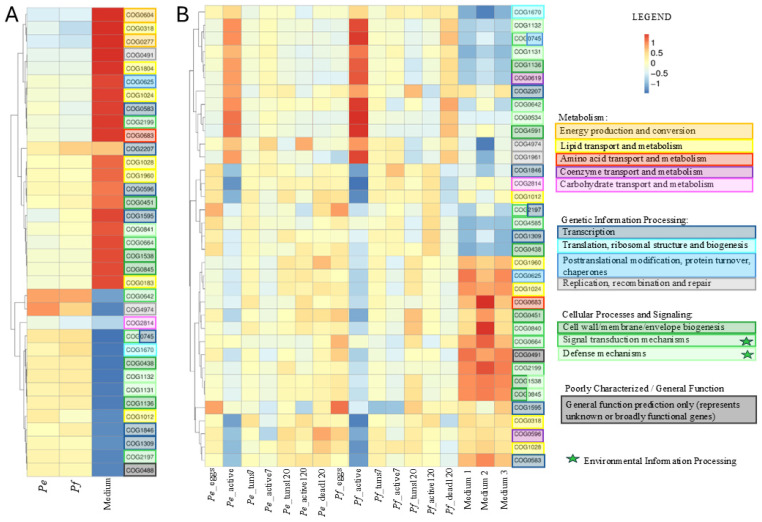
Heatmaps of functional gene (COG) profiles across samples representing stages associated with anhydrobiosis. (**A**) Comparison of relative COG abundance in *Pe* (*Paramacrobiotus experimentalis*), *Pf* (*Paramacrobiotus fairbanksi*), and the medium. (**B**) Selected functional categories across experimental variants. Colors indicate normalized values (−1 to 1; red—higher, blue—lower abundance). Dendrograms show similarity among samples and functions.

The functional profiling of microbial communities using the Clusters of Orthologous Groups (COG) database, which classifies bacterial 16S rRNA genes based on their predicted functions, was performed (Figure 7A,B). A total of 4358 COGs were identified (Table S14). To assess potential differences in the functional profiles of microbiomes associated with tardigrade species and the medium—as well as to determine whether microbial functions change across the host’s developmental and physiological stages—heatmaps were generated. Results clearly showed that COG pathways were consistent between both *Paramacrobiotus* species and significantly distinct from those in the medium. The three most abundant COGs in the tardigrade microbiomes were from the following categories: signal transduction mechanisms; transcription; and replication, recombination and repair DNA. In contrast, the most abundant COGs in the medium belonged to functional categories associated with energy production and conversion, lipid transport and metabolism and cell wall/membrane/envelope biogenesis.

After dividing the microbiome community associated with tardigrades according to developmental and physiological stages, the following patterns were observed and found to be consistent for both *Paramacrobiotus* species:

- *Pe*_eggs and *Pf*_eggs: Most COGs exhibited a slightly downregulated pattern, with only a few showing moderate upregulation. The most abundant COGs in this stage belonged to the transcription category.
- *Pe*_active and *Pf*_active: An increased abundance of all COGs grouped in the first clade of the heatmap was observed, belonging to categories such as defence mechanisms, cell wall/membrane/envelope biogenesis and coenzyme transport and metabolism. At the same time, a decreased abundance was detected for COGs associated with categories such as transcription, carbohydrate transport and metabolism and lipid transport and metabolism.
- *Pe*_tuns7, *Pf*_tuns7, *Pe*_tuns120 and *Pf*_active120: The functional profile of the microbiome in the tun stage showed distinct patterns across various COGs. These patterns were more similar to those observed in the microbiome of eggs than in active specimens.
- *Pe*_active7, *Pf*_active7, *Pe*_active120 and *Pf*_active120: The top COGs identified in the stage after anhydrobiosis were from categories, e.g., DNA replication, recombination and repair.
- *Pe*_dead120 and *Pf*_dead120: No common patterns were observed between both *Paramacrobiotus* species.

**Figure 8:**
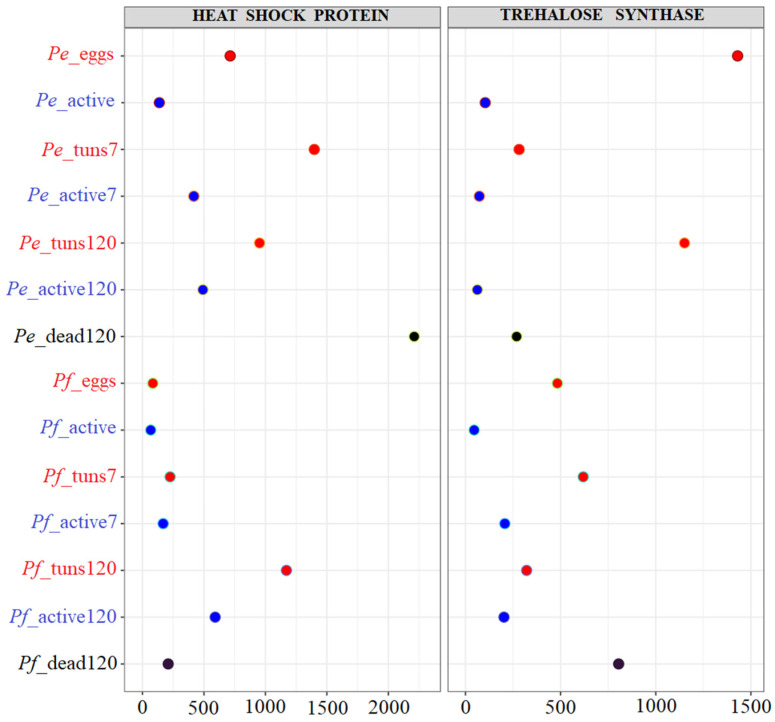
Selected COGs that showed increased predicted gene copy numbers during desiccation include heat shock protein (HslJ) and trehalose synthase (TreT).

The predicted gene copy numbers of heat shock proteins (HslJ, COG3187) and trehalose synthase (TreT, COG0380) increased during the egg (*Pe*_eggs and *Pf*_eggs) and tun (*Pe*_tuns7, *Pf*_tuns7, *Pe*_tuns120 and *Pf*_active120) stages. Moreover, phage shock protein C (PspC, COG1983) and uncharacterized proteins overlap with the distribution of HslJ and TreT (Table S14—selected COGs have been marked in blue). During active stages, both before anhydrobiosis (*Pe*_active and *Pf*_active) and after short- and long-term anhydrobiosis (*Pe*_active7, *Pf*_active7, *Pe*_active120 and *Pf*_active120), all of the above proteins and sugars showed very low or near-zero predicted gene copy numbers. In contrast, in dead specimens (*Pe*_dead120 and *Pf*_dead120), the distribution of these factors appeared random, but in most cases, they indicated genes with minimal predicted gene copy numbers (Figure 8).

## 3. Discussion

Anhydrobiotic organisms, like tardigrades, have evolved various adaptations to survive extreme water loss [2], but the exact mechanisms behind this desiccation tolerance are not fully understood. Interestingly, some molecular adaptations, such as the production of the non-reducing, cellular-component-stabilizing sugar trehalose, are shared between tardigrades and bacteria [1,22], suggesting a potential link. However, it is still unknown if the host’s microbiome plays a role in anhydrobiosis.

### 3.1. Does the Microbiome Community of Tardigrades Change During Anhydrobiosis?

In the present study, Firmicutes, Actinobacteriota and Proteobacteria were the most abundant bacterial phyla associated with *Pam. experimentalis* and *Pam. fairbanksi*. In contrast, the microbiome of the laboratory medium (with rotifers as a tardigrade food) was dominated by Proteobacteria, Actinobacteriota and Bacteroidota. These results are consistent with previous studies indicating that tardigrade microbiomes are distinct from their environment and altered by laboratory culture [9,11,13,16,17].

The microbiome community composition varied across the two *Paramacrobiotus* species’ developmental and physiological stages. Eggs exhibited the lowest alpha diversity and tun stages showed reduced diversity compared to active specimens. Tun stages further displayed unique bacterial taxa not observed in other stages. We further observed that the longer the time spent in anhydrobiosis, the lower the microbial diversity in the subsequently rehydrated specimens, indicating that a prolonged desiccation state reduces microbial richness. The most diverse microbiome community was observed in dead specimens after long anhydrobiosis, likely due to, as is known in the literature, microbial blooms or opportunistic colonization after host death [23]. Although the similar microbiome structure identified for both tardigrade species is not too surprising, considering that they belong to the same genus and were maintained under identical laboratory conditions for several dozen generations, our results clearly demonstrate that anhydrobiosis affects microbial diversity.

### 3.2. Which Core Microbiome Bacteria Potentially Influence Tardigrade Anhydrobiosis?

A core microbiome, comprising 29 OTUs, was consistently detected across developmental and physiological stages of both *Paramacrobiotus* species, indicating potential functionally important bacterial taxa. However, most of these OTUs were also identified in the medium with rotifers, but at lower frequencies suggesting that the presence of these bacteria in tardigrades may be due to horizontal transfer from the external environment. Regardless of origin, these bacteria may play an important role in anhydrobiosis, as both tardigrades and some rotifers are capable of inducing anhydrobiosis [1]. It should be kept in mind however, that the analyzed 16S rRNA gene fragment is relatively conserved and often precludes bacterial identification to the species level [24]. Consequently, sequences in the tardigrade microbiome and the surrounding medium that were classified as the same OTU may in fact represent different species. For this reason, all analyses in the present study were focused on OTUs classified at the genus level.

Bacterial genera shared between the tardigrade core microbiome and their surrounding medium raise important questions on their origin and functional relevance to tardigrade physiology, particularly anhydrobiosis. Overall, genera such as *Staphylococcus*, *Corynebacterium*, *Acidocella*, *Ralstonia*, *Streptococcus* and *Actinomyces* were abundant and widely distributed, suggesting their persistence is not incidental, but may reflect roles in host physiology. Notably, many of these bacterial genera are also commonly found as symbiotic or opportunistic partners with eukaryotic hosts. For instance, *Staphylococcus* can modulate immune responses and form protective biofilms [25], as well as adapt to extreme environments [26]. Furthermore, a survey of the microbiome of glacial tardigrades reported that animal commensals, such as *Streptococcus* species and members of the *Actinomyces* genus, were common in both fully fed and starved tardigrades, suggesting that they are core components of the tardigrade microbiome [17]. The open question is whether these bacteria play some role in the survival of tardigrades under stressful conditions.

Three OTUs were unique to the core microbiome of the tardigrades—Chitinophagaceae, Neisseriaceae and *Gemella*. Members of the Chitinophagaceae are commonly found in soil and freshwater environments and are frequently detected in the microbiome of various invertebrates, including tardigrades [9,11]. They are known for their ability to degrade complex polysaccharides such as chitin and cellulose, and for producing a range of extracellular enzymes and secondary metabolites [27]. Although a direct role in anhydrobiosis has yet to be experimentally demonstrated, several metabolic traits of Chitinophagaceae suggest a synergistic function. For instance, some species produce trehalose and/or polyols, which are known to protect cells from desiccation-induced damage by stabilizing proteins and membranes [28]. Additionally, Chitinophagaceae produce reactive oxygen species (ROS) scavengers or enzymes such as catalases and peroxidases, which could be valuable in mitigating the oxidative stress in tardigrades undergoing cycles of dehydration/rehydration [29].

Members of the bacterial family Neisseriaceae, commonly associated with mucosal surfaces in vertebrates, have also been detected in environmental and invertebrate microbiomes, including those of tardigrades [9,18]. Several members of the family have been shown to synthesize catalase, superoxide dismutase and other antioxidant enzymes [30], which may mitigate ROS damage during desiccation and rehydration. Similarly, bacteria of the genus *Gemella*, typically associated with the mucosal microbiota of humans and other vertebrates, but also identified in environmental samples and the microbiome of invertebrates, including tardigrades [9,18], possess robust oxidative stress response mechanisms, including superoxide dismutase and peroxidase systems [31]. This is particularly relevant given the strong oxidative stress response associated with anhydrobiosis in tardigrades [32].

### 3.3. Could Bacteria Associated with Microbial Shifts During Anhydrobiosis Influence This Phenomenon?

In the egg-associated microbiome of *Pam. fairbanksi*, *Ferruginibacter* was identified as a characteristic microbial member. Although this genus was also detected in the egg microbiome of *Pam. experimentalis*, statistical analysis did not classify it as a significantly characteristic taxon at that stage. Interestingly, *Ferruginibacter* species was present in the tun7 stage in both *Paramacrobiotus* species. Members of this genus, belonging to the family Chitinophagaceae, are recognized as part of the core tardigrade microbiome [11,13] and are known for their ability to degrade complex polysaccharides [33], as well as possessing genes involved in the synthesis or metabolism of osmoprotective compounds such as trehalose [34]. These features imply that members of the *Ferruginibacter* may contribute to microbiome stabilization during desiccation and possibly protect the host by preserving cellular structures or serving as a source of compatible solutes during rehydration [32]. Furthermore, its ability to form biofilms (through the production of extracellular polymeric substances (EPS)) could provide additional protection by forming a hydrated microenvironment, buffering desiccation and shielding against environmental stressors such as UV radiation, ROS and temperature fluctuations [34]. Biofilms may also act as reservoirs for protective metabolites, supporting tardigrades during rehydration. The presence of *Ferruginibacter* species in eggs and the tun7 stage suggests that it must have also been present during the active stage, but at undetectable levels. However, its absence after 120 days of anhydrobiosis may indicate that prolonged desiccation leads to its elimination or a drastic reduction in abundance. This raises the question of whether the observed loss of *Ferruginibacter* species correlates with the appearance of dead specimens, suggesting its possible essential role in successful anhydrobiosis? Interestingly, *Ferruginibacter* species was also a common bacterium associated with the glacial tardigrade *Cry. klebelsbergi*, with its frequency increasing under induced environmental stress—in this case, starvation [17]. This finding was also congruent with the microbiome community of tardigrades described by refs. [9,35].

In the microbiome of active *Pam. fairbanksi*, *Eubacterium* was more characteristic. Recent studies of tardigrade-associated microbial communities have identified members of the genus *Eubacterium* [9]. Although their presence in tardigrades may initially seem surprising, some *Eubacterium* species are metabolically comprehensive and capable of producing short-chain fatty acids (SCFAs), such as butyrate, acetate, and propionate, which are known to influence host energy metabolism, immune modulation, and stress responses [36]. In contrast, the active-stage microbiome of *Pam. experimentalis* was more strongly characterized by the presence of *Ottowia* and members of the Rikenellaceae group. *Ottowia* is commonly found in aquatic and wastewater environments and is known for its metabolic adaptability, including the ability to degrade various organic substrates under both aerobic and facultative anaerobic conditions [37]. In turn the Rikenellaceae family is often associated with complex carbohydrate degradation and SCFA production, particularly in host-associated microbiome [38].

The tun7 microbiome stage of *Pam. fairbanksi* was characterized by the presence of *Sanguibacter*-*Flavimobilis*, and *Aurantisolimonas*. These genera are known for their environmental resilience and may contribute to stress adaptation, possibly through antioxidant activity or membrane-stabilizing metabolites [39]. In contrast, *Pam. experimentalis* showed increased abundance of biofilm-forming bacteria: *Rahnella*, *Serratia*, *Aquabacterium* and *Lactobacillus*. Two of these taxa, such as *Lactobacillus* and *Serratia*, are known for producing protective compounds like exopolysaccharides and trehalose, which could assist in stabilizing host tissues or maintaining microbial viability under desiccation [40].

The microbiome of *Pam. fairbanksi* at the active7 stage was further characterized by the presence of *Schlegelella* and *Kineococcus*. *Schlegelella* is a versatile environmental bacterium capable of degrading selected organic compounds [41]. *Kineococcus* is recognized for desiccation tolerance and pigmentation that can confer resistance to oxidative stress [42]. In turn, in *Pam. experimentalis*, the microbiome of active individuals was characterized by *Moraxella* and *Gardnerella*. *Moraxella* is a genus of facultatively anaerobic bacteria often found in host-associated niches and capable of surviving fluctuating oxygen conditions [43], suggesting a potential role in early recovery dynamics post-rehydration. *Gardnerella*, typically associated with mucosal environments and biofilm formation [44], may contribute to microbial stabilization on tardigrade surfaces or within gut-like cavities during the rehydration phase. At the tun120 stage, the microbiome of *Pam. fairbanksi* was notably characterized by the presence of *Vibrionimonas*. For *Pam. experimentalis* no specific bacteria that were characteristic in the tun120 stage was detected. Overall, members of *Vibrionimonas* are typically found in aquatic and marine environments and are known for their metabolic versatility and ability to survive under nutrient-limited and stress-prone conditions [45].

Interestingly, several bacterial genera were detected exclusively in tuns (but not identified as main characteristic taxa in this stage), i.e., *Vibrionimonas* and *Lacticaseibacillus*. This could indicate their possible specialization in surviving or functioning under extreme desiccation. Indeed, *Vibrionimonas*, known for its metabolic versatility and stress tolerance in aquatic environments, may contribute to maintaining microbial stability and nutrient cycling during prolonged dormancy [46]. In turn, *Lacticaseibacillus* is recognized for producing protective metabolites such as exopolysaccharides [47]. Overall, tun-associated bacteria likely form a specialized microbiome that contributes to the tardigrade’s ability to survive extreme dehydration by maintaining host and microbial community stability through biofilm formation (e.g., *Lacticaseibacillus*, *Nocardia*, *Streptococcus*, *Acinetobacter*, *Cutibacterium* and *Micrococcus*), producing protective biomolecules such as heat shock proteins and/or trehalose (e.g., *Lacticaseibacillus*, *Corynebacterium* and *Pseudomonas*), and supporting the host’s metabolic needs during and after anhydrobiosis. In the case of *Pseudomonas*—this genus has frequently been detected in studies of the tardigrade microbiome (e.g., refs. [11,18]). In particular, Vecchi et al. [9] identified *Pseudomonas* as part of the core microbiome, while Zawierucha et al. [17] reported it as one of the dominant taxa in *Cry. klebelsbergi* collected from cryoconite holes. The genus *Pseudomonas* is highly diverse, encompassing both pathogenic and non-pathogenic species [48]. A specific *Pseudomonas* strain in *Caenorhabditis elegans* provide protection against fungal pathogens [49], which led Tibbs-Cortes et al. [16] to speculate that similar beneficial interactions may occur in tardigrades. If confirmed, such associations could indicate that components of the tardigrade microbiome contribute to host defence and environmental adaptability.

In the microbiome of *Pam. fairbanksi* at the active120 stage, the species of the genus *Massilia* were notably enriched. This genus was also detected in the microbiome of *Pam. experimentalis* at the same physiological stage, but it was not described as characteristic. Overall, members of the genus *Massilia* are widely recognized for their metabolic adaptability and resilience to various environmental stresses [50].

At the dead120 stage, a notable shift in microbial composition was observed. In *Pam. fairbanksi*, the microbiome was dominated by members of *Ornithinimicrobium*, *Cereibacter* and *Acidiphilium*. *Ornithinimicrobium*, often linked to soil and decomposing matter [51], may reflect opportunistic colonization during early decomposition. In *Pam. experimentalis*, members of the *Microbacterium* dominated dead120 specimens, indicating a shift toward decomposition-associated microbial succession [52]. The prevalence of these genera suggests microbiome deregulation and colonization by opportunistic taxa.

It should also be mentioned that although the number of replicates per developmental stage was limited to three, the statistical approach applied here remains appropriate for the ecological patterns under investigation. A replication level of n = 3 per group is widely used in multivariate community analyses and it has been shown to be sufficient for detecting consistent multivariate structure [53]. Importantly, SIMPER does not rely on large sample sizes but on the stability of multivariate dissimilarities within and among groups; in this study, dissimilarity patterns were highly consistent across replicates, indicating that the observed differences are not driven by sample-size artefacts [54]. Furthermore, permutation-based multivariate methods such as PERMANOVA, which were used here to complement the SIMPER results, are specifically designed to remain valid under low to moderate replication as long as the experimental design is balanced [55]. Taken together, these factors support the conclusion that, despite the limited number of replicates, the statistical inferences derived from the SIMPER analysis are robust and biologically meaningful.

### 3.4. Changes in the Functional Profiling of the Microbial Communities Related to Anhydrobiosis

Functional profiling of microbial communities was inferred from 16S rRNA data using phylogenetically informed predictions [56]. Although indirect, this approach assumes related taxa share similar gene content and metabolic capabilities. However, this assumption may not always be correct due to horizontal gene transfer, strain-level functional variability, and the presence of lineage-specific adaptations that cannot be captured solely on the basis of 16S rRNA phylogeny. Moreover, PICRUSt version 2.4.2 [56] based predictions rely heavily on the availability and completeness of reference genomes within public databases. Because these databases are dominated by human-associated and clinically relevant microorganisms, their representation of environmental and invertebrate-related taxa remains limited. Consequently, functional inference for tardigrade microbiomes may be affected by reduced accuracy, particularly for rare, novel, or poorly characterized microorganisms that are underrepresented in current genomic repositories [57]. Such limitations may result in incomplete or biased reconstruction of metabolic pathways and ecological functions.

Predicted gene families were annotated using the COG database, which clusters genes into conserved functional categories [57]. While such predictions may be less accurate for rare or poorly characterized taxa [58], consistent enrichment of specific COGs across developmental and physiological stages in both *Paramacrobiotus* species revealed distinct, stage-specific patterns. Functional profiles also differed between tardigrades and rotifer-containing media, reflecting OTU-level results and enabling clear stage differentiation. Importantly, these findings should be interpreted as predictive trends rather than direct evidence of microbial activity or gene expression, since PICRUSt infers potential functions rather than measuring them experimentally. Despite these limitations, observed functional shifts provide valuable ecological insight and underscore the usefulness of combining functional prediction with taxonomic analyses to better understand microbial contributions to stress adaptation. Furthermore, these findings establish a basis for future validation using shotgun metagenomics and metatranscriptomics, which would allow direct assessment of microbial functional capacity and activity [59]. The microbial functions potentially involved in anhydrobiosis, including heat shock protein (HslJ), phage shock protein C (PspC), trehalose synthase (TreT) and several uncharacterized proteins, were identified. Their predicted gene copy numbers increased markedly during egg and tun stages, suggesting induction under desiccation stress. In contrast, predicted gene copy numbers during active stages (before or after short- and long-term anhydrobiosis) were minimal, indicating these factors are unnecessary outside stress conditions. Dead specimens showed random, mostly negligible predicted gene copy numbers, consistent with metabolic shutdown. Although tardigrades synthesize their own protectants, the potential contribution of microbiota to these stress-response mechanisms remains largely unexplored.

Heat shock proteins such as HslJ, i.e., a bacterial stress-response protein linked to heat and oxidative stress adaptation, showed increased predicted gene copy numbers during egg and tun stages, indicating induction under desiccation stress. The HslJ has been primarily characterized in Gram-negative bacteria, especially within the Enterobacteriaceae and Moraxellaceae families [60]. However, in our study, the specific bacterial source of the HslJ predicted gene copy numbers could not be determined, highlighting the need for further targeted microbiome and metagenomic analyses to clarify its origin and role in the tardigrade-associated microbial community.

Phage shock protein C (PspC) also showed predicted gene copy number patterns linked to anhydrobiosis. In bacteria, the Psp system, particularly PspC, maintains membrane integrity and energy homeostasis under stress, including desiccation-related envelope damage [61]. *Acinetobacter*, enriched in eggs and tuns in our study, is known to activate this system during osmotic imbalance and membrane disruption, i.e., conditions associated with anhydrobiosis. The PspC function as a sensor and signal mediator, recruiting proteins that preserve proton motive force and limit membrane permeability [62]. While tardigrades lack canonical bacterial Psp operons, transcriptomic and proteomic evidence suggests PspC-like domains or analogous proteins involved in membrane protection during tun formation [32]. Such convergence underscores the critical role of membrane stabilization in surviving extreme water loss.

Trehalose synthase (TreT), which catalyzes the reversible conversion of UDP-glucose and glucose into trehalose [63], was also enriched during egg and tun stages. Trehalose, a non-reducing disaccharide, acts as a chemical chaperone that stabilizes proteins, membranes and nucleic acids under dehydration stress [64]. Many bacteria common in the tardigrade microbiome, such as members of the genera *Corynebacterium*, *Ralstonia* and *Pseudomonas*, synthesize trehalose in response to desiccation [65], and *Ralstonia* species have been previously detected in tardigrade species [9]. The role of trehalose in tardigrade anhydrobiosis, however, is species-specific. Some species (e.g., species of the order Parachela (Eutardigrada)) accumulate trehalose during desiccation [66], while others such as *Hypsibius exemplaris* lack canonical biosynthetic genes [67], suggesting reliance on alternative strategies like tardigrade-specific intrinsically disordered proteins (TDPs) [32]. This raises the possibility that trehalose detected in these species originates from symbiotic microbes rather than the host. Recent transcriptomic data from *Echiniscus testudo* confirmed that some species retain trehalose biosynthesis genes [68], highlighting variation across tardigrades and potential microbiome contributions to stress protection.

Interestingly, many uncharacterized proteins, including proteins involved in exopolysaccharide biosynthesis and in outer membrane biogenesis, as well as whose functions remain unknown, showed predicted gene copy number patterns overlapping with known desiccation-related genes such as HslJ, PspC and TreT. This co-distribution suggests that these proteins may be regulated in a similar manner in response to desiccation stress, implying a broader functional role in desiccation resistance. Their consistent presence alongside well-characterized stress response elements highlights the potential significance of these unannotated proteins for tardigrade survival under extreme conditions and warrants further functional investigation. However, the tardigrades’ specific proteins, i.e., CAHS, SAHS, MAHS, RvLEAM and Dsup, were detected in transcriptomes and they contribute to a general stress response and are related to anhydrobiosis [69]. Therefore, similar proteins in the microbiome and its host could have been acquired independently in tardigrades [6] and convergent evolution has been suggested [70]. Nevertheless, obtained using COG approach, many uncharacterized proteins are congruent with results obtained for tardigrades from the transcriptome analysis that revealed specific molecular pathways for stress adaptations [71].

### 3.5. Hypothesis, Limitations, and Future Directions

In this study, we propose a novel and previously unexplored hypothesis suggesting that bacteria may potentially contribute to or modulate tardigrade responses during anhydrobiosis. Importantly, we do not claim that the bacterial taxa identified in this study are directly involved in or essential for successful anhydrobiosis in tardigrades. Rather, we highlight putative microbial candidates that, based on their taxonomic identity and functions reported in the literature, may represent promising targets for future experimental validation.

This study is primarily hypothesis generating and is based on 16S rRNA-derived microbiome profiles; therefore, functional inferences remain indirect and predictive. Additional limitations include the inability to distinguish between metabolically active and inactive microbes, as well as the lack of direct experimental evidence linking specific taxa to physiological processes in the host. Moreover, PICRUSt based predictions are constrained by reference genome availability and may not fully capture the functional potential of invertebrate related or poorly characterized microorganisms.

Despite these limitations, our findings emphasize the importance of incorporating a microbiome perspective into studies of extreme stress tolerance in metazoans. Future research should focus on experimental validation of the proposed microbial candidates, including their functional roles under desiccation stress. Approaches such as shotgun metagenomics, metatranscriptomics, and controlled manipulation of microbiota (e.g., gnotobiotic or antibiotic treated systems) would be particularly valuable to test the causal relationships between microbial communities and the anhydrobiotic performance. Overall, this study provides a conceptual framework for exploring host–microbe interactions in tardigrades and their potential contribution to desiccation tolerance.

## 4. Materials and Methods

### 4.1. Tardigrada as an Animal Model to Study Anhydrobiosis Ability

Two *Paramacrobiotus* species kept in laboratory cultures were selected for this study:

- Specimens of *Pam. experimentalis* were collected from a moss sample on soil collected near Fort-Voyron, Antananarivo, Antananarivo Province, Madagascar (18°55′35″ S, 47°31′23″ E, 1340 m asl) in November 2013. These specimens were used to establish laboratory cultures. Tardigrades were cultured following the methodology outlined by Roszkowska et al. [72]. Briefly, males and females were maintained in a medium composed of double-distilled water and Żywiec spring water at a 3:1 ratio in sandpaper-scratched Petri dishes. Cultures were kept in a climate-controlled chamber with a 12 h light/dark cycle at 20 °C and 40% relative humidity. Rotifers (*Lecane inermis*) were provided as food *ad libitum* every week.
- Specimens of *Pam. fairbanksi* were collected from a moss sample on stone near the east end of Louise Lake, Banff National Park, Alberta, Canada (51°24′21″ N, 116°14′27″ W, 1900 m asl) in May 2019. The culture procedure was analogous to that of *Pam. experimentalis*; however, *Pam. fairbanksi* is parthenogenetic so laboratory cultures consisted of only females.

### 4.2. Rotifera Cultures

For the experiments, rotifers *Lecane inermis* from the collection of the Aquatic Ecosystems Team of the Institute of Environmental Sciences of the Jagiellonian University were used. The clone 1.A2.15 was obtained from a single specimen isolated from an activated sludge sample originating from a municipal Wastewater Treatment Plant (50°47′12″ N 18°59′47″ E) in the south of Poland. Cultures of the clone were constantly maintained in Petri dishes in a medium of Żywiec spring water in darkness at 20 ± 1 °C. Rotifers were fed with NOVO (a patented nutrition powder used for rotifer mass culture) [73].

### 4.3. Anhydrobiosis Protocol

All experiments were conducted in vented plastic Petri dishes (35 mm diameter) lined with white filter paper (grammage 85–87, Chemland Company, Starogard, Poland). Specimens were transferred into the dishes using an automatic pipette. Subsequently, dishes were placed in a climate-controlled chamber (PolLab, Q-Cell 140, Warsaw, Poland) and allowed to dry slowly in the dark at 20 °C with 40% relative humidity. The formation of tuns was monitored every 24 h using a stereomicroscope Olympus SZ51 (Olympus Corporation, Tokyo, Japan).

**Figure 9:**
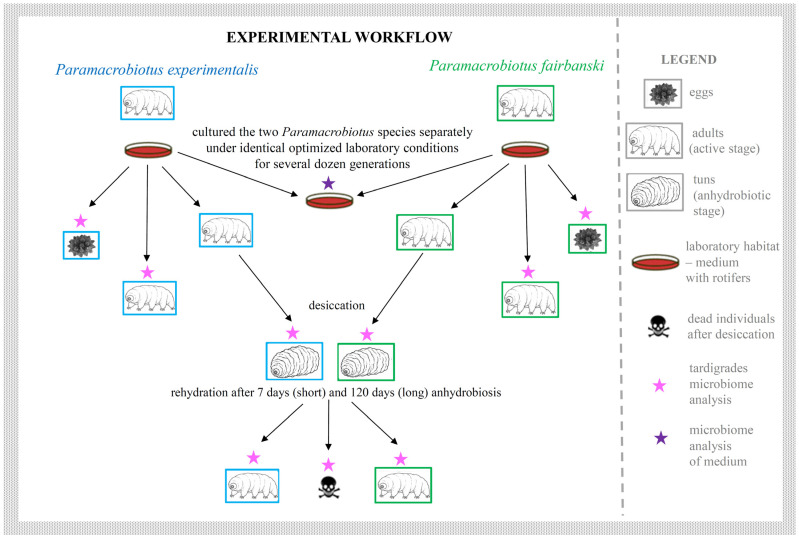
Workflow of the experimental approach.

Microbiome profiles were examined twice, i.e., after 7 days (“short anhydrobiosis”) and 120 days (“long anhydrobiosis”) in anhydrobiosis, because after this period an increased time for return to the active state had been observed [74] and could be caused by changes in microbiome communities. Upon tun formation, specimens underwent two durations of anhydrobiosis. For short-term anhydrobiosis, tuns were analyzed after 7 days, and active animals were assessed after 24 h of rehydration. No dead specimens were observed. For long-term anhydrobiosis, tuns were analyzed after 120 days, and active or dead animals were evaluated after 24 h of rehydration. Each experimental condition was replicated ten times to assess consistency and accuracy of the results and samples in different developmental and physiological stages, including tuns, which were used in microbiome community analyses (Figure 9).

The following abbreviations were applied in all analyses: *Pe*—*Pam. experimentalis*, *Pf*—*Pam. fairbanski*, 7—7 days (short) anhydrobiosis, 120—120 days (long) anhydrobiosis.

### 4.4. DNA Extraction and Amplicon Library Generation

Prior to DNA extraction for microbiome analysis, eggs and adults (i.e., fully active, non-moulting *Pam. experimentalis* and *Pam. fairbanksi* specimens) were selected from cultures. Tardigrades in the active stage were starved for three days before experiments. To minimize contamination, several steps were implemented and detailed descriptions of all procedures are provided in the section below: *Approaches to avoid contamination in tardigrades microbiome analysis*.

For DNA extraction, three samples from each stage were used, including single individuals and ten eggs per stage. DNA isolation for both species was performed at different times: for *Pam. experimentalis* in 2023 and for *Pam. fairbanksi* in 2024. Individuals representing each analyzed developmental and physiological stage were subjected to DNA extraction immediately upon reaching that respective stage. The aim was to verify the accuracy and reproducibility of the obtained results.

Additionally, negative control samples were included in three replicates: (i) to identify potential laboratory contaminants—blank samples (PCRs without a DNA template) and DNA extraction blanks; and (ii) to identify potential contaminants originating from food—the laboratory culture medium common to both *Paramacrobiotus* species was analyzed in three replicates, i.e., a 20 µL sample containing *L. inermis* specimens and their medium (hereafter referred to simply as the “medium”).

The DNA isolation for all samples was performed using the Xpure^TM^ Cell & Tissue Micro kit (A&A Biotechnology, Gdańsk, Poland, https://www.aabiot.com/xpure-cell-and-tissue-micro) accessed on 5 May 2026. To enhance microbial community lysis, some modifications to the manufacturer’s instructions were applied. Specifically, the mutanolysin and lysozyme treatment step was incorporated, as described in the Genomic Mini AX Bacteria+ Spin protocol (A&A Biotechnology, https://www.aabiot.com/en/genomic-mini-ax-bacteria-plus-spin) accessed on 5 May 2026, to ensure efficient lysis of bacteria that are particularly resistant to disruption (e.g., *Lacobacillus*, *Lactococcus*, *Listeria*, *Streptococcus*). Additionally, Eppendorf tubes were continuously shaken (500 RPM, Eppendorf Thermomixer compact 5350, Hamburg, Germany) and incubated overnight at 37 °C and then for 2 h at 50 °C. The subsequent steps were performed according to the manufacturer’s protocols. Extracted DNA was quantified using Eppendorf BioPhotometer D30 and stored at −20 °C for further analyses.

A genomic DNA isolation kit intended for small quantities of cell cultures, tissues, cell lines, microbiome and small invertebrates was used. The kit proved suitable for the isolation of DNA also from rotifers contained in 20 µL of medium, as it is optimized for very low biomass samples and efficiently recovers high-quality DNA from minute amounts of biological material.

The prokaryotic 16S rRNA molecular marker, covering 470 bp of the hypervariable V3-V4 region, was amplified using the following primer set: 341F: 5′-CCTAYGGGRBGCASCAG-3′ and 806R: 5′-GGACTACNNGGGTATCTAAT-3′. Primers were tailed-ligated with unique barcode sequences at the 5′ ends for sequencing (Table S15). All polymerase chain reactions (PCR) were carried out in a 30 μL volume containing 15 μL JumpStart Taq ReadyMix, 3 μL of the appropriate forward and reverse primers each, and 4 μL of DNA. The PCRs were performed in a Bio-Rad T100 Thermal Cycler (Bio-Rad Laboratories, Inc., San Francisco, CA, USA). The PCR cycling profile used to amplify the V3-V4 region of the 16S rRNA gene fragment was as follows: initial denaturation at 95 °C for 5 min, followed by 35 cycles of 95 °C for 30 s, 47 °C for 45 s, and 72 °C for 1 min 30 s, with a final extension at 72 °C for 7 min. For each PCR reaction, 5 μL was electrophoresed on a 1% agarose gel to assess amplification efficiency. Negative control samples of medium and blank PCRs were also amplified. When primers with standard cleaning were used, bands were observed in the blank PCRs on the agarose gel. The amplification was then repeated using primers with HPLC cleaning, and no bands were visible (Figure S2).

The PCR products were purified using magnetic beads, pooled and analyzed for target bands using Qubit and real-time PCR. An amplicon metagenetic library was prepared for PCR products that met the following criteria: appropriate purity (OD260/280 = 1.8–2.0) as well as no degradation and no detectable contamination (including verification using negative and positive controls to minimize the risk of cross-contamination). Quantified libraries were sequenced based on the required data amount and effective library concentration (>10,000 reads per sample).

### 4.5. High-Throughput 16S rRNA Amplicon Sequencing

Pooled amplicons with adaptor sequences were sequenced using the NEBNext^®^ Ultra^TM^ II DNA Library Prep Kit (Cat No. E7645) and the Illumina NovaSeq 6000 Sequencing System (Novogene, Cambridge, UK).

Paired-end reads were assigned to PCR amplicons based on their unique adaptor sequences using Python v3.6.13. Adaptors and primer sequences were removed using Cutadapt v3.3 and paired-end reads were merged with FLASH v1.2.11 [75]. To obtain high-quality Clean Tags [76], data filtration was performed using Fastp v0.23.1. Tags were compared against the Silva Database [77] to detect chimeric sequences, which were subsequently removed using the Vsearch package v2.16.0 [78]. All sequence data were processed using the QIIME 2 pipeline [79], which applies a denoising approach to resolve amplicon sequence variants with high accuracy. Prior to downstream analyses, chimeric, mitochondrial, and chloroplast sequences were removed to retain only high-quality reads of microbial origin. The resulting dataset was used for all diversity and multivariate analyses described above. Complete read counts for all samples were provided in Tables S4–S13 to ensure full transparency and reproducibility of the workflow.

### 4.6. Approaches to Avoid Contamination in Tardigrades Microbiome Analysis

Contamination is a persistent and often underestimated challenge in microbiota research, particularly in studies involving low-biomass organisms such as tardigrades. Our findings, in line with those of Surmacz et al. [18], underscore the importance of rigorous contamination control at every stage of experimental design, from sample preparation to data analysis. Nevertheless, we acknowledge that, despite best efforts, minor laboratory contamination during sequencing can never be entirely excluded, even in our study. However, we took extensive precautions to minimize this risk. Our data clearly show distinct microbiome profiles corresponding to specific developmental and physiological stages of tardigrades, as well as differences between the microbiota of the animals and that of their rotifer-enriched medium. Based on this, we are confident that our contamination control measures were effective and that the microbiome patterns we report reflect genuine biological variation.

To minimize contamination as much as possible, we implemented several strategies commonly used in microbiome studies, based on previously published protocols. These included: (i) washing tardigrades in sterile water prior to DNA extraction, as described in Vecchi et al. [9], Kaczmarek et al. [11], Mioduchowska et al. [13], Tibbs-Cortes et al. [16], Zawierucha et al. [17], Mioduchowska et al. [14], and Surmacz et al. [18]; (ii) using sterile laboratory equipment and consumables throughout all procedures (e.g., ref. [11]); (iii) extracting DNA with increased efficiency kit for genomic DNA purification (with some modifications described in Material and Methods section) and performed extraction DNA under a laminar flow chamber, as recommended for low-biomass studies (e.g., Zawierucha et al. [17]); (iv) applying three types of negative controls, following RIDE recommendations [80]: (a) a blank template (PCRs without a DNA template) (e.g., ref. [13]), (b) extraction blanks (e.g., ref. [18]) and (c) environmental controls, i.e., culture medium with rotifers (e.g., ref. [11]); (v) starving specimens prior to DNA extraction (to minimize gut content-derived bacteria (e.g., ref. [17]). In addition, we took the following steps to further reduce contamination: (i) instead of sterilizing water in-house, we used ultra-pure water from a commercial source (A&A Biotechnology); (ii) all reagents for DNA extraction and PCR were freshly purchased, and tubes were only opened immediately before use; (iii) We protected pipettes and the samples from contamination using only sterile filtered pipette tips; and (iv) we used HPLC-purified primers for 16S rRNA amplification. This step proved critical: primers purified by standard desalting (Genomed, Warsaw, Poland) resulted in visible PCR bands in negative controls after gel electrophoresis, suggesting contamination. In contrast, primers purified using HPLC (also from Genomed) eliminated these bands, indicating successful removal of background DNA (see Figure S2). However, none of the PCR and extraction blank negative controls passed quality control prior to sequencing since the number of reads was from 0 to 1537 (see Table S16). Nevertheless, we sequenced all of them and confirmed that negative controls were negligible compared to those observed in the experimental samples. Therefore, the contribution of contaminant DNA can be considered marginal and unlikely to affect the biological interpretation of the results. Overall, these findings confirm that the microbial profiles obtained from the target samples are reliable and not driven by background contamination.

### 4.7. Bioinformatics and Statistical Analysis Pipeline

The Uparse software v7.0.1001 [81] was used for sequence analysis. Sequences were clustered into the same operational taxonomic units (OTUs). Taxonomic annotation of the representative sequences for the detected OTUs was performed using the Silva Database and based on the Mothur algorithm. The MUSCLE software v3.8.31 [82] was used to perform multiple sequence alignment.

Sequence data were normalized, and the relative abundance of the top 10 taxa at the genus rank was selected to generate a distribution histogram in Perl using the SVG function. In R, the heatmap function was applied to generate a heatmap with the abundance information of the top 35 taxa from each sample, visually displaying differences in abundance and taxa clustering. Additionally, petal diagrams illustrating the common and unique OTUs among defined groups/samples were generated in R using the PetalDiagram function.

Alpha diversity was assessed using six indices: observed species; Chao1 and ACE—allowing community richness estimation; Shannon and Simpson—allowing community diversity estimation; and Good’s coverage—allowing for sequencing depth characterization. These indices were calculated using QIIME 2 and visualized in R v4.0.3. A species accumulation boxplot was generated by applying the vegan package in R [83] to evaluate microbial community richness and sample size. Additionally, a rarefaction curve was generated in R using the plyr library [84] to determine whether the appropriate sequencing depth had been achieved. In turn, beta diversity analysis was conducted to assess differences in species complexity among samples. Beta diversity, based on both weighted and unweighted UniFrac metrics, was calculated using QIIME 2.

To examine the differentiation in the microbiome composition across developmental and physiological stages, as well as between two *Paramacrobiotus* species, non-parametric multivariate tests (ANOSIM and PERMANOVA) were performed. Firstly, a two-way PERMANOVA was applied to assess whether the composition of the microbiome differed significantly between the studied species and between developmental and physiological stages. The model incorporated the main effects of both variables (species and stages) and the interaction between the factors (species × stages). The analysis was performed based on the Bray–Curtis distance matrix, and statistical significance was assessed based on 9999 permutations, with a significance level of *p* = 0.05. Secondly, one-way ANOSIM analysis was used to confirm differences in microbiome structure between groups (at different developmental and physiological stages). The R-statistic was calculated based on the Bray–Curtis distance matrix, and the significance of the differences was assessed using permutations (n = 9999). Subsequently, a similarity percentages (SIMPER) analysis was performed to identify the representatives of the microbial communities that contribute most to the observed differences (or similarities) between all of the developmental and physiological stages of *Pam. experimentalis* and *Pam. fairbanksi* described above. The average contribution of each microbial genus to the overall group difference and its percentage contribution to the differences in the Bray–Curtis dissimilarity matrix were also summarized. Furthermore, an indicator species analysis (IndVal%) was performed to determine which microbial genus most effectively characterized the individual sample groups. All multivariate statistics were performed using PAST v. 4.17 [85].

### 4.8. R Scripts

The R scripts are available at the following GitHub repository version 3.17.5 (https://github.com/bayubeta/paramacrobiotus, accessed on 9 August 2025). The first script (heatmap_availables.R) finds OTUs that appeared in all stages by only including OTUs that have a non-zero number of sequences value for every stage and arranging them in decreasing order according to their Euclidean distance to a vector of zeros, which can be expressed as:

$$d\left(x_{1},\ldots ,x_{14}\right)=\sqrt{\sum_{i=1}^{14}x_{i}^{2}}\;,$$

where $x_{i}$ is the number of sequences at the stage i of the OTU x. It was denoted $x_{1},\ldots ,\;x_{7}$ as the whole seven stages for *Pam. experimentalis* and $x_{8},\ldots ,\;x_{14}$ for *Pam. fairbanksi*.

The second script (heatmap_tun.R) investigates OTUs that increase during the tun stages. Separate analyses for the microbiome of *Pam. experimentalis* and *Pam. fairbanksi* were performed and we computed the average number of sequences in both tun stages (7- and 120-day anhydrobiosis) and the average number of sequences in all other stages, for each OTU. Only OTUs with a higher average number of sequences in tun stages than in the other stages were included. The OTUs were then sorted by their average number of sequences in tun stages, from highest to lowest.

### 4.9. Functional Prediction

Metabolic capacities were performed using PICRUSt software v1.1.4 (Phylogenetic Investigation of Communities by Reconstruction of Unobserved States [58]), which is primarily used to infer metagenetic functions based on the 16S rRNA structure of the microbiota. Representative OTU sequences derived from experimental data were normalized for 16S rRNA gene copy numbers and metagenomes were predicted. Clusters of Orthologous Groups (COGs) were identified using the NCBI pipeline [86]. The COG repertoire of each sample was defined as the number of unique COGs annotated in the respective gene set(s), irrespective of the number of genes assigned to each COG or whether a single gene was annotated to multiple COGs.

The PICRUSt database is commonly used for functional prediction; however, it should be note that it has certain limitations for invertebrate microbiomes, as its reference genomes are mainly based on human-associated microbes.

## 5. Conclusions

This study revealed that anhydrobiosis is accompanied by pronounced shifts in microbiome composition and predicted functional profiles in two *Paramacrobiotus* species. Distinct microbial communities were associated with eggs, active specimens, and tun stages, indicating strong stage-specific structuring under desiccation stress.

Bacterial taxa enriched during tun and egg stages were linked in the literature to traits such as oxidative stress protection, biofilm formation and osmoprotectant production, suggesting that the microbiome may represent an additional component of the tardigrade stress response system.

Altogether, these findings provide the first evidence that microbiome dynamics are closely associated with anhydrobiosis in tardigrades and establish a basis for future studies on host–microbe interactions under extreme environmental conditions.

## Data Availability

The R scripts are available at the following GitHub repository version 3.17.5 (https://github.com/bayubeta/paramacrobiotus, accessed on 9 August 2025). In turn, bacterial 16S reads for each sample were submitted to the NCBI BioProject database under submission number PRJNA1310788.

## Supplementary Materials

The following supporting information can be downloaded at: https://www.mdpi.com/article/10.3390/ijms27125256/s1.

## Abbreviations

The following abbreviations are used in this manuscript:

*Pe*	*Pam. experimentalis*
*Pf*	*Pam. fairbanski*
7	7 days (short) anhydrobiosis
120	120 days (long) anhydrobiosis
